# Pamidronate treatment in resistant cases of CRMO – our small case series

**DOI:** 10.1186/1546-0096-12-S1-P247

**Published:** 2014-09-17

**Authors:** Eva Vrtíková, Elena Košková, Tomáš Dallos

**Affiliations:** 1 National Institute of Rheumatic Diseases, Piešťany, Slovakia; 2 2nd Department of Paediatrics, Comenius University Medical School, Bratislava, Slovakia

## Introduction

Chronic recurrent multifocal osteomyelitis (CRMO) is the most common autoinflammatory osteopathy. CRMO is characterized by non-bacterial osteomyelitis and primarily affects children and adolescents. The disorder has a relapsing and remitting course. While non-steroidal anti-inflammatory drugs (NSAIDs) are used as first-line treatment, glucocorticoids and disease-modifying antirheumatic drugs have been reported to be effective in some cases. In resistant cases, the effects of TNF-α inhibitors or bisphosphonates were observed.

## Objectives

To describe a cohort of CRMO patients and to characterize treatment effects with focus on bisphosphonates in a retrospective study of a case series.

## Methods

Between 1995 and 2014 we diagnosed CRMO in 17 patients (14 females and 2 males). The mean age at diagnosis was 9.2 years. The time between symptom onset and diagnosis of CRMO ranged from 3 to 72 months. We found multilocal lesions involving the tibia, clavicle, femur, vertebral bodies, pelvis and mandible. The average number of lesions per patient was 3.8. The diagnosis of CRMO was based on radiographic findings (X-ray, CT, MRI, scintigraphy) and biopsy. In the histopathology are typical cell infiltrates – predominantly neutrophils in the early stages, monocytes, macrophages, lymphocytes and plasma cells in the later stages; and osteolyses with sclerosis and/or fibrosis in late CRMO phases. After confirming CRMO all patients were treated with NSAID.

## Results

NSAID was ineffective in 8 out of 17 patients who then received a corticosteroid therapy. 4 of these patients (3 girls and 1 boy) did not fully respond and were treated with intravenous pamidronate. In 2 patients pamidronate was added to MTX and/or Sulphasalzine (n=2) while the other 2 patients were MTX/Sulphasalazine naive.

In MTX/Sulphasalazine naive patients, 1 reached complete remission after a single dose and 1 after a double dose in a 6 month regimen. In these patients no corticosteroids were needed after pamidronate therapy.

Two patients with previous MTX/Sulphasalazine treatment, the dosing regimen at 6-month intervals was inadequate, requiring a dose increase to 3-month intervals. One of these patients was treated with anti-TNF-α inhibitors prior to pamidronate, but without sufficient effect. These patients have improved only partially and required treatment with corticosteroids. In both groups no adverse effects were observed.

## Conclusion

In accordance with published data, our experience confirms good effect of pamidronate therapy in patients with resistant CRMO, however more severe cases with multiple bone lesions and/or systemic symptoms (febrile, inflammatory skin disorders, weight loss or growth retardation) require higher dose regimen.

## Disclosure of interest

None declared.

